# Genetic Polymorphisms Associated with the Neutrophil–Lymphocyte Ratio and Their Clinical Implications for Metabolic Risk Factors

**DOI:** 10.3390/jcm7080204

**Published:** 2018-08-08

**Authors:** Boram Park, Eun Kyung Choe, Hae Yeon Kang, Eunsoon Shin, Sangwoo Lee, Sungho Won

**Affiliations:** 1 Department of Public Health Sciences, Seoul National University, Seoul 08826, Korea; 90may11@gmail.com (B.P.); sunghow@gmail.com (S.W.); 2 Department of Surgery, Seoul National University Hospital Healthcare System Gangnam Center, Seoul 06236, Korea; 3 Department of Internal Medicine, Seoul National University Hospital Healthcare System Gangnam Center, Seoul 06236, Korea; joybell77@hanmail.net; 4 DNALink, Inc., Seoul 03759, Korea; esshin@dnalink.com; 5 Samsung Electronics, Inc., Suwon 16677, Korea; lsw00kor@gmail.com; 6 Institute of Health and Environment, Seoul National University, Seoul 08826, Korea

**Keywords:** neutrophil, lymphocyte, metabolic risks, polymorphism

## Abstract

Background: The neutrophil–lymphocyte ratio (NLR) is a valuable prognostic or predictive biomarker in various diseases, but the genetic factors that underlie the NLR have not been studied. We attempted to investigate polymorphisms related to NLR phenotype and analyze their ability to predict metabolic risks. Methods: A genome-wide association study was performed with log-transformed NLR using an Affymetrix Axiom™ KORV1.1-96 Array. Regression models for metabolic risk status were designed using the identified significant single-nucleotide polymorphisms (SNPs). Results: We identified four SNPs near the *TMEM116*, *NAA25*, and *PTPN11* genes that were associated with the NLR. The top SNP associated with the log-transformed NLR was rs76181728 in *TMEM116*. A case–control study was performed to analyze the metabolic risks associated with each SNP after adjusting for age, sex, and body mass index (BMI). Three SNPs displayed significant odds ratios (ORs) for increased blood pressure and increased waist circumference. In the regression model for metabolic syndrome, rs76181728 showed a significant association (OR = 1.465, 95% confidence interval (CI) = 1.091–1.969, *P* = 0.011) after adjustment for the NLR phenotype. Conclusions: We identified four novel SNPs that are associated with the NLR in healthy Koreans. SNPs in relevant genes might therefore serve as biomarkers for metabolic risks.

## 1. Introduction

Inflammatory status plays an important role in determining the pathophysiology and prognosis of various diseases. Recently, the neutrophil–lymphocyte ratio (NLR) has been explored as a biomarker of inflammation [1]. The NLR has previously been reported as a prognostic or predictive biomarker in a variety of cancers [2,3,4], metabolic diseases [5], and cardiovascular diseases [6,7,8]. In a meta-analysis of renal cell carcinoma, a higher NLR predicted poorer overall survival (hazard ratio, 1.82) and poorer recurrence-free/progression-free survival (hazard ratio, 2.18) [3]. In a study performed in an Asian Indian population, the NLR was higher in metabolic syndrome, and as the number of metabolic abnormalities decreased, the NLR also decreased in a linear manner [5]. Finally, in a cohort representative of the general population in the United States, the NLR was found to be a predictor of cardiovascular mortality and was used to accurately reclassify patients at intermediate risk of cardiovascular disease according to their Framingham risk score [8].

The NLR represents the balance between leukocytes and neutrophils, and expresses how dominant neutrophils are over lymphocytes. Modulation of the NLR may therefore reflect a deeper imbalance in the immunological responses [1]. This notion is supported by the observation that the NLR is associated with the propagation of various cytokines [1]. However, the genetic mechanisms underlying how the NLR is modulated have not been studied. We propose that NLR-associated genetic factors could affect a variety of diseases before an NLR phenotype manifests. In this study, we evaluated two outcome measures. First, we performed a genome-wide association study (GWAS) to identify genetic polymorphisms that are related to the NLR phenotype. Second, using the results obtained from the GWAS of the NLR phenotype, we investigated the clinical implications of the identified single-nucleotide polymorphisms (SNPs). We chose metabolic syndrome as a representative condition in this analysis. Metabolic syndrome is a disease that has been reported to be related to the NLR level and has a large influence on healthcare. This condition is estimated to affect one quarter of the world’s adult population, and 28.8% of general population have it [1]. We sought to determine whether the polymorphisms identified in this study can be used to predict metabolic syndrome or metabolic risk factors in addition to the impact of NLR phenotype.

## 2. Methods

### 2.1. Study Subjects

We retrospectively used clinical and genetic data stored between 2014 and 2015 in the Gene–Environmental Interaction and Phenotype (GENIE) database for Koreans. The details of the characteristics and structure of the GENIE study are described elsewhere [9]. Briefly, DNA samples were collected from peripheral blood obtained from individuals who participated in comprehensive health checkups. SNP genotyping was performed using an Affymetrix Axiom™ KORV1.1-96 Array (Thermo Fisher Scientific, Santa Clara, CA, USA) by DNA Link, Inc. From this database, individuals for whom blood neutrophil and lymphocyte counts were available in their health checkup data were included. Individuals with a history of malignancy, acute inflammatory conditions, or infectious diseases were excluded.

### 2.2. Clinical Assessment and Definitions

All blood samples were obtained after the subjects had fasted for at least 10 h. To diagnose metabolic syndrome, we followed the criteria suggested by the American Heart Association [10], which requires at least three of the following metabolic risks: increased waist circumference (males >102 cm; females >88 cm); elevated triglycerides (≥150 mg/dL); reduced high-density lipoprotein (HDL) cholesterol (males <40 mg/dL; females <50 mg/dL); elevated fasting glucose (fasting glucose ≥100 mg/dL or the use of medications for hyperglycemia); and elevated blood pressure (≥130/85 mmHg or the use of medications for hypertension). The consumption of more than 140 g of alcohol per week was regarded as a yes in the questionnaire. The subjects were grouped as non-smokers/ex-smokers vs. current smokers.

### 2.3. Ethics Statement

The Institutional Review Board of the Seoul National University Hospital approved this study protocol (H-1512-055-727) and waived the need for informed consent. The study was performed in accordance with the Declaration of Helsinki.

### 2.4. Genotyping, Quality Control and Imputation

We performed systematic quality control in the procedure described below as presented elsewhere [11]. Samples matching the following criteria were excluded: sex inconsistencies; call rate up to 97%; and related- or cryptically-related individuals (identical by state (IBS) >90%). SNPs were filtered out if the minor allele frequency was approximately 5%, the excessive missing genotype was more than 3%, or there was a significant deviation from the Hardy–Weinberg equilibrium permutation test (*P* < 10^−5^). We also excluded SNPs that were likely to indicate false positive associations due to incorrect clustering. After quality control was performed, 345,072 SNPs remained.

GWAS imputation was carried out using SHAPEIT2 v2.r837 [12] and IMPUTE2 [13] version 2.3.2 for pre-phasing the data and genotype imputations. We used 1000 Genomes Phase 3 haplotypes as the reference panel. The imputation accuracies of the analyzed SNPs were confirmed with an info metric obtained from IMPUTE2, and SNPs less than 0.5 INFO were removed. After imputation, a total of 3,693,205 SNPs from 7303 individuals were used for the analysis. A population stratification analysis was performed using principal component analysis (Figure S1) to assess the influence of race using the EIGENSIFT package version 6.1.4.

### 2.5. Statistical Analysis

The NLRs were log-transformed to approximate a normal distribution. We categorized the NLR into normal NLRs (<mean NLR + 1 standard deviation) vs. increased NLR (≥mean NLR + 1 standard deviation) based on an arbitrary cut-off value (mean NLR +1 standard deviation). The calculated cut-off value was 2.84. We used multiple regression models to determine the associations between the log-transformed NLRs and the SNPs in the PLINK software packages, version 1.9 [14]. Principal component scores were estimated with WISARD software (http://statgen.snu.ac.kr/wisard/). Age, sex, and body mass index (BMI) were used as covariates in the additive model. The results were verified using discovery and validation sets. Subjects enrolled between January 2014 and April 2015 were included in the discovery set (*n* = 4986), and subjects enrolled between May 2015 and December 2015 were included in the validation set (*n* = 2271). SNPs that fulfilled the Bonferroni corrected *P*-value, 5×10^−8^, in the discovery set were re-evaluated in the validation set. *P*-values less than 0.05 were considered statistically significant in the validation set.

We also analyzed the associations of log-transformed white blood cell (WBC) counts, neutrophil counts, and lymphocyte counts with SNPs because the NLR is related to these parameters.

Using a multiple logistic regression analysis, we performed a case–control study for each metabolic risk factor according to the significant SNPs in additive models after adjusting for age, sex, and BMI. Odds ratios (ORs), 95% confidence intervals (CIs), and corresponding *P*-values were calculated for candidate SNPs.

Finally, from the discovery data, we designed various regression models for metabolic risk evaluation. We used the most significant SNP according to dominant genotypic modeling in the multiple logistic regression analysis after adjusting for various factors, such as age, sex and BMI in model 1; age, sex, smoking status, alcohol consumption, and BMI for model 2; age, sex, BMI, and log-transformed NLR for model 3; and age, sex, smoking status, alcohol consumption, BMI, and log-transformed NLR for model 4.

The R statistical software (Version 3.4.4) package was used for statistical analyses, and *P*-values less than 0.05 were considered significant.

## 3. Results

### 3.1. Characteristics of the Study Population

The characteristics of the subjects in the discovery and validation sets are described in Table 1. 

A total of 7257 healthy individuals underwent regular health checkups, including white blood cell count, neutrophil, and lymphocyte counts. The mean age was 50.5 ± 10.2 years old, and 4208 (58%) of the subjects were men. The mean NLR was 1.9 ± 0.9, and 10% of the population was in the increased NLR category. Metabolic syndrome was detected in 17.9% of the population. Based on previously described methods, 4986 subjects were classified into the discovery set, and 2270 were classified into the validation set. A quantile–quantile (Q-Q) plot is shown in Figure S2.

### 3.2. Genome-Wide Association Study of the Neutrophil–Lymphocyte Ratio

**Figure 1:**
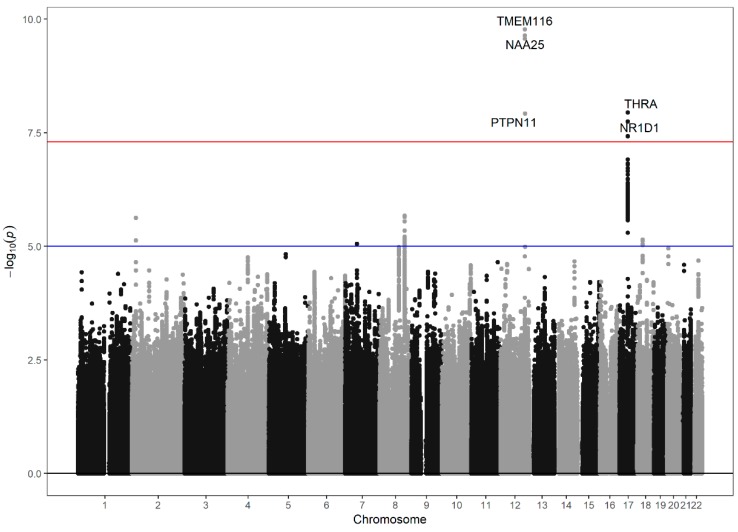
Manhattan plot of the strength of association [−log10 (*P*) values; Y axis] between single-nucleotide polymorphisms (SNPs) (X-axis by chromosome and chromosomal position) and log-transformed NLR levels. Threshold line: 5 × 10^−8^.

First, we carried out a GWAS in the discovery set with *P*-values below 5 × 10^−8^ used as the threshold for significance after adjusting for age, sex, and BMI. In the discovery set, 9 SNPs were significantly associated with the log-transformed NLR. Among these 9 SNPs, 5 SNPs were imputed, and 4 SNPs were originally genotyped. The estimated imputation accuracies for the imputed SNPs were all greater than 0.9, and their INFO values are represented in Table S1. We selected these SNPs for additional study in the validation set and found that all were significant (*P*-values less than 0.05, Table S1). Figure 1 shows the Manhattan plot of the GWAS of log-transformed NLR levels. Table S2 shows the linkage disequilibrium data for the significant SNPs (Figure S3). After these analyses, the SNPs were grouped into four regions: rs76596471, rs76181728, rs79945097 and rs7977554 near the *TMEM116* gene, *NAA25*, and *PTPN11* in chromosome 12; rs62065216, rs7502233, and rs7502539 near the *THRA* gene in chromosome 17; rs1879265 near the *THRA* gene in chromosome 17; and rs2102928 near the *NR1D1* gene in chromosome 17.

Second, we performed the same GWAS for WBC counts, lymphocyte counts, and neutrophil counts because the NLR is a function of neutrophil and lymphocyte counts, both of which are included in the total WBC count. Hence, the association between NLR phenotype and significant SNPs might actually be related to the WBC, lymphocyte, or neutrophil status. The imputed SNPs rs76181728, rs76596471, rs7977554 and genotyped SNPs rs79945097 displayed a single-handed association with the NLR (Table S3). These four SNPs are located on chromosome 12 and were associated with the *TMEM116*, N-alpha-acetyltransferase 25, NatB auxiliary subunit (*NAA25*), and *PTPN11* genes. Regarding linkage disequilibrium, all four SNPs had pairwise R2 values greater than 9.90 and D values greater than 0.98. A regional plot was obtained for the SNP rs76181728, which was most highly associated with the log-transformed NLR (Figure S4).

### 3.3. Case–Control Study for the Presence of Metabolic Syndrome According to the 9 Significant SNPs

We performed a case–control study using an additive model for each metabolic risk and metabolic syndrome with 9 SNPs that were significantly association with log-transformed NLRs when adjusted for age, sex, and BMI. Among the nine significant SNPs, three (rs76181728, rs79945097, and rs76596471) with a single-handed association with the NLR, as shown above, had a significant OR for increased blood pressure and increased waist circumference. Although these results did not reach statistical significance, the SNPs displayed a tendency toward an association with metabolic syndrome (Table 2).

None of the SNPs was significantly associated with increased triglycerides, decreased HDL cholesterol or elevated fasting glucose (Table S4).

### 3.4. Regression Models for Increased Blood Pressure, Decreased HDL Cholesterol and Metabolic Syndromes

The top SNP associated with the log-transformed NLRs was rs76181728 in the *TMEM116* gene (*P*-value, discovery set = 1.68 × 10^−10^; validation set = 0.00749). We designed several regression models for increased blood pressure, decreased HDL cholesterol, and metabolic syndromes using various risk factors including rs76181728 as the dominant genotypic model (Table 3).

The results showed that rs76181728 was significantly associated with increased blood pressure (OR = 1.308, 95% CI = 1.072–1.597, *P* = 0.008) in model 1 (age-, sex- and BMI- adjusted). The SNP was also significantly associated (OR = 1.342, 95% CI = 1.098–1.641, *P* = 0.004) after additional adjustment for the NLR phenotype (log-transformed NLR), as shown in model 3. All four models resulted in a significant OR for predicting increased waist circumference. Regression model 4 had the highest OR for increased waist circumference (OR = 1.563, 95% CI = 1.114–2.193, *P* = 0.009) and was constructed using rs76181728 as the dominant genotype adjusted for age, sex, smoking status, alcohol consumption, BMI, and log-transformed NLR.

For metabolic syndrome, rs76181728 had a significant OR of 1.418 (95% confidence interval = 1.057–1.901, *P* = 0.019) in model 2 and showed a significant association (OR = 1.465, 95% CI = 1.091–1.969, *P* = 0.011) after additional adjustment for the NLR phenotype (log-transformed NLR) in model 4.

## 4. Discussion

This is the first GWAS to evaluate the NLR, and our results indicate that four novel SNPs, rs76181728, rs79945097, rs76596471 and rs7977554, are associated with the NLR. These markers are associated with three genes, *PTPN11*, *NAA25*, and *TMEM116*, that are located on chromosome 12. rs76181728, the most significant of the four SNPs, had the power to predict metabolic risks such as increased waist circumference, increased blood pressure and metabolic syndrome after adjustment for NLR phenotype (log-transformed NLR).

The NLR, which represents the balance between neutrophils and lymphocytes in the body, is a biomarker of systemic inflammation and has recently been the subject of substantial amount of research [15]. The NLR reflects processes in two different immune pathways: neutrophils reflect ongoing immune responses, while lymphocytes reflect immune regulatory pathways [16,17]. This balance between neutrophils and lymphocytes has been suggested to reflect the deep-seated immune status [1].

However, the pathophysiological processes that underlie disruptions to the balance between neutrophils and lymphocytes have not been identified, and the corresponding genetic background has not been reported. In this GWAS, nine SNPs were found to be associated with the NLR. As shown in Table S3, SNPs that were significantly associated with either neutrophil or lymphocyte counts were excluded so that only those SNPs that were significantly associated with the NLR were selected for analysis. Thus, only novel SNPs that are uniquely associated with NLR were identified.

We selected rs76181728, which had the highest significance among the identified novel SNPs, to construct a regression model for metabolic syndrome and metabolic risks. In future studies, further analyses could potentially use this marker to predict metabolic syndrome. We found that rs76181728 was independently associated with waist circumference, blood pressure and metabolic syndrome.

Interestingly, rs76181728 maintained its independent predictive power even when the models applied were adjusted for NLR phenotype (log-transformed NLR). Though the NLR phenotype is known to predict metabolic risks on its own, information related to this polymorphism may provide an additional effect for predicting metabolic risks. Therefore, SNPs associated with the NLR can be used as biomarker for metabolic syndrome or its associated risks, for which the NLR phenotype has previously been used as a biomarker. Even before the NLR phenotype manifests, SNPs associated with the NLR can be used as biomarkers to predict various diseases, such as metabolic syndrome and related risks. However, our findings should be replicated in other populations possessing different characteristics and applied to the analysis of cancer or cardiovascular disease regression models.

The novel SNPs identified in our study were associated with three genes, namely, *PTPN11*, *NAA25*, and *TMEM116*. The *PTPN11* gene encodes protein tyrosine phosphatase non-receptor type 11, also known as protein tyrosine phosphatase 1D, SHP-2 [18,19]. Protein tyrosine phosphatases are involved in signaling pathways that regulate cellular activation, proliferation and differentiation [20]. These signaling events are important for mitogenic activation, metabolic control, and cell migration [21,22]. Evidence from a mouse model showed that SHP-2 regulates glucose and lipid metabolism [23], and in aged mice, hepatocyte-specific deletion of SHP-2 promoted inflammatory signaling and hepatic inflammation/necrosis [24]. Activating SHP-2 mutations have been observed in neuroblastoma, melanoma, breast cancer, lung cancer, and colorectal cancer [25]. Some GWASs have demonstrated an association between the *PTPN11* gene and gastric cancer [26], colitis, and serum lipid levels [27,28]. Considering that there are pathophysiological associations between the *PTPN11* gene and the diseases described above, *PTPN11* could be involved in a mechanism that connects the NLR to inflammation, cancer and metabolic diseases.

The *NAA25* gene encodes NAA25, which functions in normal cell cycle progression [29] and cell cycle regulation [30]. The *TMEM116* gene encodes transmembrane protein 116, which has been reported to be associated with atrial fibrillation [31] and diabetes [32].

Linkage disequilibrium at the 12q24 locus has been hypothesized to interfere with genes such as *CUTL2*, *FAM109A*, *SH2B3*, *ATXN2*, *BRAP*, *ACAD10*, *ALDH2*, *MAPKAPK5*, *TMEM116*, *ERP29* [9], *NAA25/C12orf30*, *TRAFD1*, *HECTD4*/*C12orf51*, *RPL6*, and *PTPN11* [32,33,34,35], including the genes we identified by the nine novel SNPs. The 12q24 locus in humans has been suggested to be associated with susceptibilities to various diseases and conditions [32], such as hematopoietic diseases [36,37,38], type 1 diabetes [33,34,39,40], rheumatoid arthritis [41,42], abnormal serum urate levels [43], hypertension [35,44], and obesity [45]. Given that all of the SNPs identified here were found to be associated with genes located at the 12q24 locus, the pathophysiology underlying and predictive function of the NLR in various diseases is likely to be associated with the status of the 12q24 locus. This phenomenon should be further investigated in biochemical studies and replicated in larger populations.

Our study has several limitations. First, we enrolled individuals undergoing self-paid comprehensive health checkups. Therefore, the socioeconomic characteristics of the populations was relatively good, and these individuals may be healthier than those in the average population. Second, we performed statistical analyses in discovery and validation sets but did not have a population for replication. Thus, these analyses should be repeated in a larger population with different characteristics. Third, all of the enrolled individuals were Korean. Our findings should therefore be replicated in populations with other ethnicities in future studies. Fourth, because the SNPs we discovered are novel, no previous papers have reported on their function. In future studies, experiments should be carried out to analyze the association between the novel SNPs and the protein expression of their related genes. Fifth, the clinical implications of the discovered SNPs should be evaluated in a larger variety of diseases, such as cancer and cardiovascular diseases. In this study, we included only metabolic syndrome in our analysis. It is our intention to collect more information in a broader variety of phenotypes in future studies. Sixth, blood parameters such as monocytes, lymphocyte–monocyte ratio, and neutrophil–monocyte ratio reflect the immune status. In this study, we did not collect the monocyte count during the data collection process. To elucidate the comprehensive effect of the genetic factors of myeloid compartments on various diseases, those parameters should be investigated further.

In conclusion, we identified novel SNPs that are associated with the NLR and showed that the most significant SNPs might be useful for predicting increased waist circumference, increased blood pressure, and metabolic syndrome even after adjustment for the NLR phenotype. Therefore, SNPs in the relevant genes at locus 12q24 might have a potential to be used as biomarkers for diseases that have previously been predicted only by the NLR phenotype. The predictive value of these SNPs as biomarkers should be further evaluated in various diseases, and the results should be replicated in other populations.

## Figures and Tables

**Table 1:** Baseline characteristics of the study population.

	Total	Discovery Set	Validation Set	
*N*	(*N* = 7257)	(*N* = 4986)	(*N* = 2271)	*P*
Age	50.5 ± 10.2	50.6 ± 10.4	50.2 ± 9.7	0.081
NLR	1.9 ± 0.9	1.9 ± 0.9	1.9 ± 0.9	0.075
* NLR				0.230
Normal	6657 (91.7%)	4487 (90.0%)	2022 (89.0%)	
Increased	600 (8.3%)	499 (10.0%)	249 (11.0%)	
WBC count (×10^3^/μL)	5.4 ± 1.5	5.3 ± 1.5	5.5 ± 1.5	0.001
Neutrophil count (×10^3^/μL)	3.1 ± 1.2	3.1 ± 1.2	3.2 ± 1.2	0.001
Lymphocyte count (×10^3^/μL)	1.7 ± 0.5	1.7 ± 0.5	1.7 ± 0.5	0.018
High sensitivity C-reactive protein	0.1 ± 0.3	0.1 ± 0.3	0.1 ± 0.4	0.127
Gender				0.007
Men	4208 (58.0%)	2838 (56.9%)	1370 (60.3%)	
Women	3049 (42.0%)	2148 (43.1%)	901 (39.7%)	
BMI	23.1 ± 3.0	23.1 ± 3.0	23.3 ± 3.0	0.009
Smoking				0.985
None or ex-smoker	3196 (50.4%)	2184 (50.5%)	1012 (50.3%)	
Current smoker	1997 (31.5%)	1363 (31.5%)	634 (31.5%)	
Missing	1149 (18.1%)	782 (18.1%)	367 (18.2%)	
Alcohol consumption				0.026
No	4583 (63.2%)	3161 (63.4%)	1422 (62.6%)	
Yes	1559 (21.5%)	1033 (20.7%)	526 (23.2%)	
Missing	1115 (15.4%)	792 (15.9%)	323 (14.2%)	
Diabetes medication				0.482
No	6896 (95.2%)	4744 (95.3%)	2152 (94.9%)	
Yes	350 (4.8%)	234 (4.7%)	116 (5.1%)	
Hypertension medication				0.640
No	6030 (83.2%)	4150 (83.4%)	1880 (82.9%)	
Yes	1216 (16.8%)	828 (16.6%)	388 (17.1%)	
Waist circumference	82.6 ± 8.8	82.6 ± 8.8	82.5 ± 8.7	0.668
Triglyceride	107.9 ± 72.4	107.1 ± 72.1	109.6 ± 73.3	0.172
High-density lipoprotein cholesterol	53.9 ± 12.0	53.8 ± 12.1	53.9 ± 12.0	0.709
Fasting glucose	98.2 ± 16.6	97.7 ± 15.6	99.5 ± 18.5	0.000
Systolic blood pressure	115.4 ± 13.2	115.2 ± 13.4	115.7 ± 12.9	0.198
Diastolic blood pressure	75.9 ± 10.3	75.6 ± 10.4	76.6 ± 10.0	0.000
Metabolic syndrome	1320 (18.2%)	890 (17.9%)	430 (19.0%)	0.284

* We categorized NLR into normal NLR (<mean NLR + 1 standard deviation) vs. increased NLR (≥ mean NLR + 1 standard deviation) according to an arbitrary cut-off value (mean NLR + 1 standard deviation). The calculated cut-off value was 2.84. NLR: neutrophil–lymphocyte ratio; BMI: body mass index; WBC: white blood cell.

**Table 2:** Case–control study to evaluate the associations among the significant SNPs and hypertension, increased waist circumference, and metabolic syndrome. OR: odds ratio; CI: confidence interval.

SNP	^a^ Elevated Blood Pressure			^b^ Increased Waist Circumference			Metabolic Syndrome		
	OR	95% CI	*P*-Value	OR	95% CI	*P*-Value	OR	95% CI	*P*-Value
rs76181728	1.348	1.111–1.635	0.002	1.42	1.063–1.897	0.017	1.253	0.972–1.615	0.082
rs79945097	1.348	1.112–1.635	0.002	1.424	1.067–1.901	0.016	1.263	0.98–1.626	0.071
rs76596471	1.341	1.105–1.627	0.003	1.429	1.071–1.908	0.015	1.266	0.983–1.631	0.068
rs7977554	1.209	0.987–1.48	0.067	1.44	1.071–1.935	0.016	1.178	0.901–1.54	0.231
rs7502539	1.072	0.974–1.181	0.156	1.05	0.907–1.214	0.515	1.09	0.96–1.237	0.184
rs7502233	1.07	0.972–1.179	0.167	1.057	0.914–1.222	0.458	1.093	0.963–1.24	0.168
rs1879265	1.046	0.95–1.15	0.361	0.929	0.805–1.073	0.318	0.89	0.784–1.009	0.069
rs62065216	1.111	1.013–1.219	0.025	0.984	0.856–1.131	0.822	0.993	0.88–1.121	0.912
rs2102928	1.007	0.878–1.155	0.918	0.964	0.785–1.183	0.724	0.956	0.799–1.143	0.621

Adjusted for age, sex, and BMI * Additive models were used for genotyping SNPs. ^a^ Elevated blood pressure (blood pressure ≥ 130/85 mmHg or use of medications for hypertension). ^b^ Increased waist circumference (males > 102 cm; females > 88 cm).

**Table 3:** Regression models for metabolic risks and metabolic syndrome with the SNP rs76181728 used as the dominant genotype.

Predicting Feature	^a^ Model 1	^b^ Model 2	^c^ Model 3	^d^ Model 4
	OR (95% CI), *P*-Value	OR (95% CI), *P*-Value	OR (95% CI), *P*-Value	OR (95% CI), *P*-Value
^e^ Increased NLR	0.584 (0.413–0.827), 0.002	0.616 (0.415–0.913), 0.015		
Increased blood pressure	1.308 (1.072–1.597), 0.008	1.169 (0.928–1.474), 0.186	1.342 (1.098–1.641), 0.004	1.204 (0.954–1.52), 0.118
Increased waist circumference	1.418 (1.054–1.908), 0.021	1.506 (1.075–2.11), 0.017	1.427 (1.059–1.924), 0.019	1.563 (1.114–2.193), 0.009
Metabolic syndrome	1.242 (0.957–1.612), 0.103	1.418 (1.057–1.901), 0.019	1.27 (0.977–1.651), 0.073	1.465 (1.091–1.969), 0.011

**^a^** Model 1: adjusted for age, sex and BMI. **^b^** Model 2: adjusted for age, sex, smoking status, alcohol consumption and BMI. **^c^** Model 3: adjusted for age, sex, BMI and log-transformed NLR. **^d^** Model 4: adjusted for age, sex, smoking status, alcohol consumption, BMI, and log-transformed NLR. **^e^** Normal NLR: NLR < mean NLR + 1 standard deviation vs. Increased NLR: NLR ≥ mean NLR + 1 standard deviation. Dominant models were used for genotyping SNPs. For comparisons of the regression models, the reference was the model that used the AA or AG allele (minor alleles), and the ORs were calculated by comparison with the GG allele (the major homologous allele).

## Supplementary Materials

The following are available online at http://www.mdpi.com/2077-0383/7/8/204/s1, Table S1: SNPs associated with the log-transformed NLR, Table S2: Linkage disequilibrium among the discovered SNPs, Table S3: SNPs associated with log-transformed NLR, WBC count, lymphocyte count, and neutrophil count, Table S4: Case–control study of each metabolic risk according to the significant SNP, Figure S1: Principal component analysis (PCA) to adjust the population stratification, Figure S2: Q-Q plot of the log-transformed NLR genome-wide association study, Figure S3: Linkage disequilibrium plot among significant SNPs, Figure S4: Regional plot for the top SNP rs76181728 associated with the log-transformed NLR.
