# Supplementary material for: Genetic Polymorphisms Associated with the Neutrophil–Lymphocyte Ratio and Their Clinical Implications for Metabolic Risk Factors
Source: J Clin Med. 2018 Aug 8;7(8):204. doi: 10.3390/jcm7080204 (PMC6111840; doi:10.3390/jcm7080204)
Supplement: Supplementary file 1 [file jcm-07-00204-s001.pdf]

Supplementary materials: Genetic Polymorphisms Associated with the Neutrophil-Lymphocyte Ratio and Their Clinical Implications for Metabolic Risk Factors

Table S1. SNPs associated with the log-transformed NLR.

						Discovery							Validation						
**SNP**	**Ch**	**Position**	**Nearest gene**	**Minor/ major allele**	**INFO**	**MAF**	**Mean NLR**			**Beta**	**95% CI (lower, upper)**	**P**	**MAF**	**Mean NLR**			**Beta**	**CI**	***P* value**
							**DD**	**DR**	**RR**					**DD**	**DR**	**RR**		**95% CI (lower, upper)**	
rs76181728	12	112437124	TMEM116	A/G	0.997	0.058	1.924	1.715	1.479	−0.111	−0.145, −0.077	1.68x10^{-10}	0.056	1.957	1.813	1.73	−0.062	−0.111, −0.012	0.00749
rs79945097	12	112470241	NAA25	A/G	1	0.059	1.924	1.717	1.479	−0.11	−0.144, −0.076	2.29x10^{-10}	0.057	1.957	1.813	1.781	−0.060	−0.109, −0.011	0.00837
rs76596471	12	112412518	TMEM116	C/T	0.999	0.058	1.923	1.717	1.479	−0.11	−0.144, −0.076	2.68x10^{-10}	0.056	1.957	1.816	1.73	−0.060	−0.11, −0.011	0.00843
rs7977554	12	112882859	PTPN11	A/G	0.990	0.055	1.922	1.725	1.595	−0.103	−0.138, −0.068	1.19x10^{-8}	0.055	1.955	1.853	1.666	−0.044	−.095, 0.006	0.04135
rs7502539	17	38219005	THRA	A/G	0.997	0.337	1.96	1.866	1.79	−0.049	−0.065, −0.032	1.13x10^{-8}	0.349	1.986	1.925	1.861	−0.023	−0.048, 0.001	0.03012
rs7502233	17	38218804	THRA	G/A	1	0.34	1.96	1.868	1.79	−0.048	−0.064,	1.79x10^{-8}	0.351	1.988	1.922	1.855	−0.025	−0.05,	0.020375

											−0.031							−0.001	
rs1879265	17	38231376	THRA	G/A	0.993	0.387	1.967	1.879	1.796	−0.046	−0.062, −0.03	$3.73x10^{-8}$	0.398	1.965	1.948	1.87	−0.022	−0.046, 0.002	0.033725
rs62065216	17	38218773	THRA	G/A	1	0.463	1.967	1.899	1.811	−0.044	−0.06, −0.029	$3.77x10^{-8}$	0.475	1.991	1.942	1.88	−0.027	−0.05, −0.004	0.01076
rs2102928	17	38253228	NR1D1	T/C	1	0.13	1.936	1.791	1.729	−0.066	−0.089, −0.042	$3.79x10^{-8}$	0.133	1.966	1.853	2.123	−0.032	−0.066, 0.002	0.03254

* Adjusted for age, sex and BMI. [##] Mean NLR: back-transformed value of log-transformed NLR. [##] *P* values are based on the linear regression analysis of the log-transformed NLR and adjusted for age and sex. An additive genetic model was used.

Table S2. Linkage disequilibrium among the discovered SNPs.

SNP				
**L1**	**L2**	**D**	**R2**	**Distance**
rs76596471	rs76181728	1	0.998	24606
rs76596471	rs79945097	0.998	0.996	57723
rs76596471	rs7977554	0.988	0.94	470341
rs76181728	rs79945097	1	0.998	33117
rs76181728	rs7977554	0.988	0.94	445735
rs79945097	rs7977554	0.988	0.942	412618
rs62065216	rs7502233	0.992	0.586	31
rs62065216	rs7502539	0.999	0.591	232
rs62065216	rs1879265	0.902	0.595	12603
rs62065216	rs2102928	0.881	0.135	34455
rs7502233	rs7502539	0.999	0.994	201

rs7502233	rs1879265	0.655	0.35	12572
rs7502233	rs2102928	0.874	0.222	34424
rs7502539	rs1879265	0.656	0.35	12371
rs7502539	rs2102928	0.872	0.222	34223
rs1879265	rs2102928	0.939	0.209	21852

Table S3. SNPs associated with log-transformed NLR, WBC count, lymphocyte count, and neutrophil count.

		log NLR	log neutrophil count	log WBC count	log lymphocyte count
**SNP**	**Set**	**Beta (95% CI);** ***P* value**	**Beta (95% CI);** ***P* value**	**Beta (95% CI);** ***P* value**	**Beta (95% CI);** ***P* value**
rs76181728	Total	−0.095 (−0.123—0.067); 2.58510^{-11}	−0.045 (−0.069—0.02); 3.511 x10^{-7}	−0.009 (−0.028—0.008); 0.284	0.051 (0.031—0.070); 2.873x10^{-7}
	Discovery	−0.111 (−0.145—0.077); 1.68x10^{-10}	−0.05756 (−0.087—0.028); 1.41x10^{-4}	−0.0158 (−0.038—0.006); 0.15	0.0532 (0.03—0.077); 7.76x10^{-6}
	Validation	−0.062 (−0.111—0.012); 0.007	−0.0157 (−0.06—0.028); 0.24	0.004313 (−0.028—0.037); 0.4	0.046 (0.012—0.08); 4.49x10^{-3}
rs79945097	Total	−0.094 (−0.122—0.066); 4.184 x10^{-11}	−0.043 (−0.068—0.019); 5.103 x10^{-3}	−0.00909 (−0.027—0.009); 0.322	0.0505 (0.031—0.07); 2.527 x10^{-7}
	Discovery	−0.11 (−0.144—0.076); 2.29x10^{-10}	−0.05631 (−0.086—0.027); 1.90x10^{-4}	−0.01506 (−0.037—0.007); 0.17	0.0534 (0.03—0.077); 6.87x10^{-6}
	Validation	−0.060 (−0.109—0.011): ; 0.008	−0.01449 (−0.058—0.029); 0.26	0.004827 (−0.027—0.037); 0.38	0.0455 (0.011—0.08); 4.45x10^{-3}
rs76596471	Total	−0.09402 (−0.122—0.066); 4.687 x10^{-11}	−0.0439 (−0.068—0.019); 4.575 x10^{--4}	−0.00946 (−0.028—0.009); 0.304	0.05015 (0.031—0.069); 3.416 x10^{-7}
	Discovery	−0.11 (−0.144—0.076);	−0.05612 (−0.086—0.027);	−0.01485 (−0.037—0.007);	0.0535 (0.03—0.077);

		$2.68x10^{-10}$	$2.04x10^{-4}$	0.18	$7.02x10^{-6}$
	Validation	−0.060 (−0.11—0.011); 0.008	−0.0162 (−0.06—0.028); 0.24	0.003392 (−0.029—0.036); 0.42	0.0443 (0.01—0.079); $5.82x10^{-3}$
rs7977554	Total	−0.08378 (−0.113—0.055); $1.356x10^{-8}$	−0.04239 (−0.068—0.017); $1.065x10^{-3}$	−0.01154 (−0.03—0.007); 0.226	0.04141 (0.021—0.061); $4.771\ x10^{-5}$
	Discovery	−0.103 (−0.138—0.068); $1.19x10^{-8}$	−0.05544 (−0.086—0.025); $4.43x10^{-4}$	−0.01588 (−0.039—0.007); 0.17	0.0476 (0.023—0.072); $1.30x10^{-4}$
	Validation	−0.044 (−0.095—0.006); 0.041	−0.01561 (−0.06—0.029); 0.25	−0.002621 (−0.036—0.03); 0.44	0.0288 (−0.006—0.064); 0.05
rs7502539	Total	−0.04055 (−0.054—0.027); $6.94\ x10^{-9}$	−0.042 (−0.054—0.03); $1.012\ x10^{-11}$	−0.0257 (−0.034—0.017); $1.232\ x10^{-8}$	−0.001 (−0.011—0.008); 0.807
	Discovery	0.049 (−0.065—0.032); $1.13x10^{-8}$	−0.0504 (−0.065—0.036); $9.82x10^{-12}$	−0.03001 (−0.041—0.019); $3.22x10^{-8}$	−0.0019 (−0.013—0.01); 0.75
	Validation	−0.023 (−0.048—0.001); 0.030	−0.02355 (−0.045—0.002); 0.02	−0.01685 (−0.033—0.001); 0.02	$-3.00x10^{-4}$ (−0.017—0.017); 0.49
rs7502233	Total	−0.0407 (−0.054—0.027); $5.867\ x10^{-9}$	−0.04204 (−0.054—0.03); $6.656\ x10^{-12}$	−0.0259 (−0.035—0.017); $8.768\ x10^{-9}$	−0.001 (−0.011—0.008); 0.7794
	Discovery	−0.048 (−0.064—0.031); $1.79x10^{-8}$	−0.05044 (−0.065—0.036); $8.72x10^{-12}$	−0.0303 (−0.041—0.02); $2.26x10^{-8}$	−0.0026 (−0.014—0.009); 0.65
	Validation	−0.025 (−0.05—0.001); 0.020	−0.02438 (−0.046—0.003); 0.01	−0.01686 (−0.033—0.001); 0.02	$9.00x10^{-4}$ (−0.016—0.018); 0.46
rs1879265	Total	−0.038 (−0.052—0.025); $2.648\ x10^{-8}$	−0.03899 (−0.051—0.027); $8.850\ x10^{-11}$	−0.02372 (−0.032—0.015); $7.702\ x10^{-8}$	−0.001 (−0.01—0.009); 0.892
	Discovery	−0.046 (−0.062—0.03); $3.73x10^{-8}$	−0.04892 (−0.063—0.035); $1.54x10^{-11}$	−0.03017 (−0.041—0.02); $1.36x10^{-8}$	−0.0028 (−0.014—0.008); 0.62
	Validation	−0.022 (−0.046—0.002); 0.034	−0.01792 (−0.039—0.003);0.05	−0.01006 (−0.026—0.005);0.1	0.0043 (−0.012—0.021);

					0.3
rs62065216	Total	−0.039 (−0.052—0.026); 5.17 x10^{-9}	−0.04252 (−0.054—0.031); 2.421 x10^{-13}	−0.02658 (−0.035—0.018); 4.704 x10^{-10}	−0.003775 (−0.013—0.005); 0.409
	Discovery	−0.044 (−0.06—0.029); 3.77x10^{-8}	−0.05157 (−0.065—0.038); 1.95x10^{-13}	−0.03262 (−0.043—0.023); 2.31x10^{-10}	−0.0072 (−0.018—0.004); 0.19
	Validation	−0.027 (−0.05—0.004); 0.011	−0.0237 (−0.044—0.003); 0.01	−0.01424 (−0.029—0.001); 0.03	0.0032 (−0.013—0.019); 0.35
rs2102928	Total	−0.05522 (−0.075—0.036); 2.108 x10^{-8}	−0.04849 (−0.065—0.032); 1.969 x10^{-8}	−0.02621 (−0.039—0.014); 3.632 x10^{-5}	0.00674 (−0.007—0.02); 0.3208
	Discovery	−0.066 (−0.089—0.042); 3.79x10^{-8}	−0.05796 (−0.078—0.038); 2.77x10^{-8}	−0.03134 (−0.046—0.016); 4.21x10^{-5}	0.0079 (−0.008—0.024); 0.34
	Validation	−0.032 (−0.066—0.002); 0.033	−0.02795 (−0.058—0.002); 0.03	−0.0154 (−0.038—0.007); 0.09	0.0041 (−0.019—0.028); 0.37

* Adjusted for age, sex and BMI. * *P* values are based on the linear regression analysis of the log-transformed NLR and adjusted for age and sex. An additive genetic model was used.

Table S4. Case-control study of each metabolic risk according to the significant SNP.

SNP	[a] Elevated triglycerides			[b] Reduced HDL cholesterol			[c] Elevated fasting glucose		
	OR	95% CI	*P* value	OR	95% CI	*P* value	OR	95% CI	*P* value
rs76181728	1.098	0.868~1.389	0.434	0.906	0.715~1.149	0.416	1.054	0.866~1.284	0.599
rs79945097	1.092	0.863~1.381	0.462	0.923	0.73~1.168	0.504	1.047	0.86~1.275	0.65
rs76596471	1.11	0.878~1.404	0.383	0.907	0.716~1.15	0.421	1.061	0.871~1.292	0.555
rs7977554	1.098	0.859~1.403	0.456	0.833	0.646~1.074	0.158	1.022	0.832~1.255	0.835
rs7502539	0.953	0.848~1.07	0.415	0.953	0.85~1.069	0.409	1.019	0.926~1.123	0.696
rs7502233	0.957	0.853~1.075	0.462	0.958	0.855~1.074	0.465	1.019	0.925~1.122	0.706
rs1879265	0.918	0.818~1.029	0.143	0.893	0.798~1	0.05	0.955	0.868~1.051	0.349

rs62065216	0.935	0.837~1.044	0.231	0.906	0.813~1.01	0.075	0.975	0.889~1.069	0.593
rs2102928	0.912	0.773~1.076	0.273	0.931	0.791~1.095	0.388	0.988	0.862~1.133	0.862

* Adjusted for age, sex and BMI. * Additive models were used for genotyping SNPs. [a] Elevated triglycerides (triglycerides ≥150 mg/dL). [b] Reduced high-density lipoprotein (HDL) cholesterol (males <40 mg/dL; females <50 mg/dL). [c] Elevated fasting glucose (fasting glucose ≥100 mg/dL or use of medications for hyperglycemia).

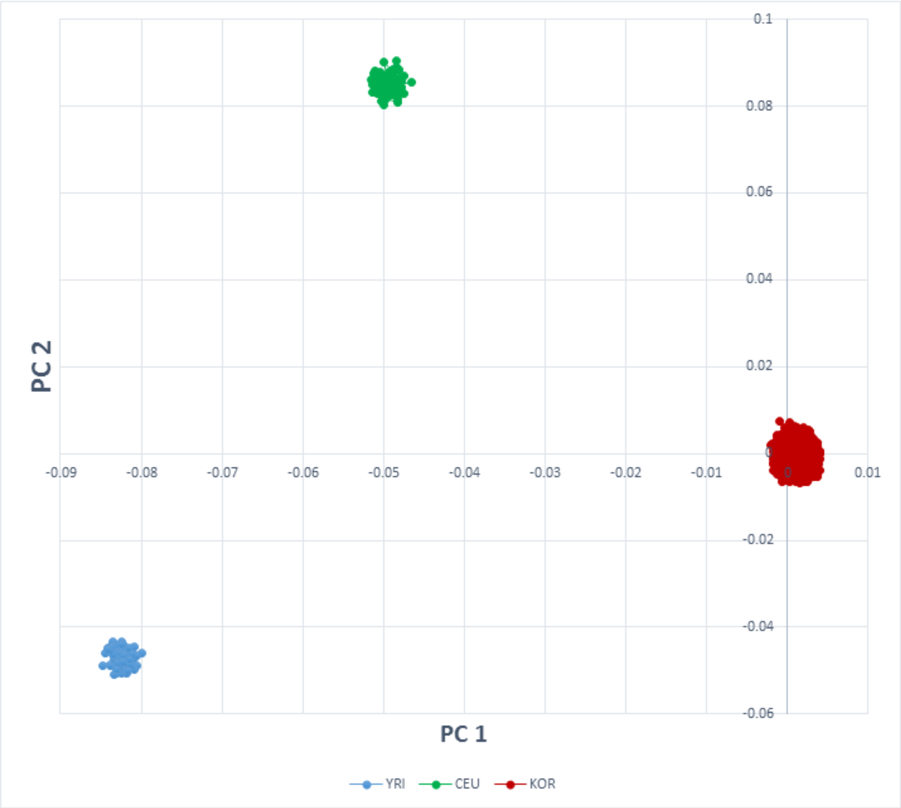

Figure S1. Principle component analysis (PCA) to adjust the population stratification. The total population of the GENIE database[9] was merged with the YRI and CEU data, which consisted of 1000 genomes, for the PCA. The criteria for marker selections were as follows: quality control of minor allele frequencies >0.05, call rates >0.05, Hardy-Weinberg equilibrium (*P* > 0.0001), and autosome. In all, 220,222 overlapping markers were found among the datasets. We randomly selected 20% of the overlapping markers (43,979) for the PCA plot.

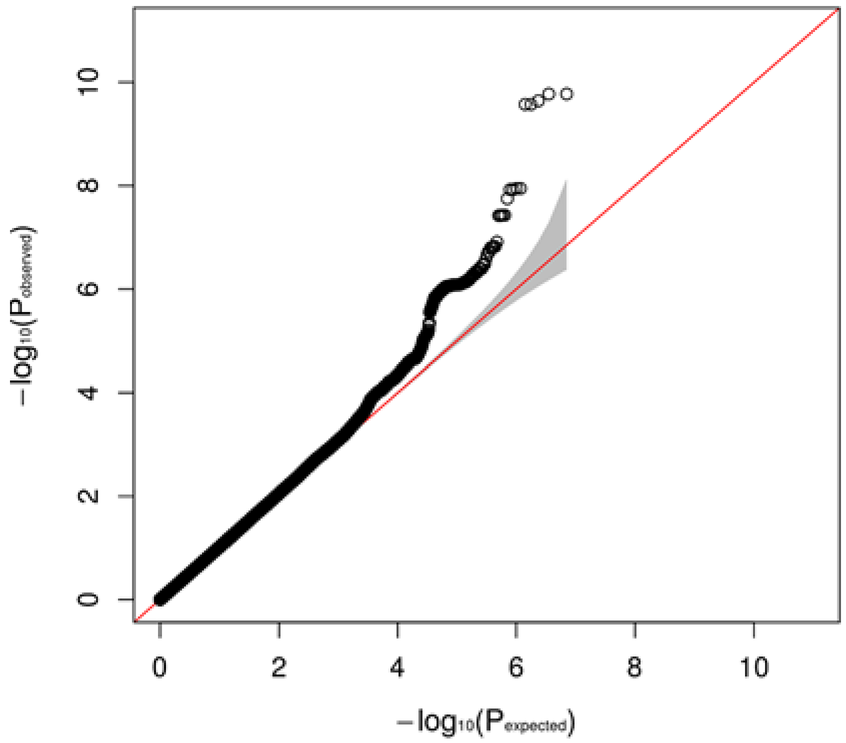

Figure S2. Q-Q plot of the log-transformed NLR genome-wide association study. The variance inflation factor (VIF) is 1.02, which suggests that the type-1 error is well controlled.

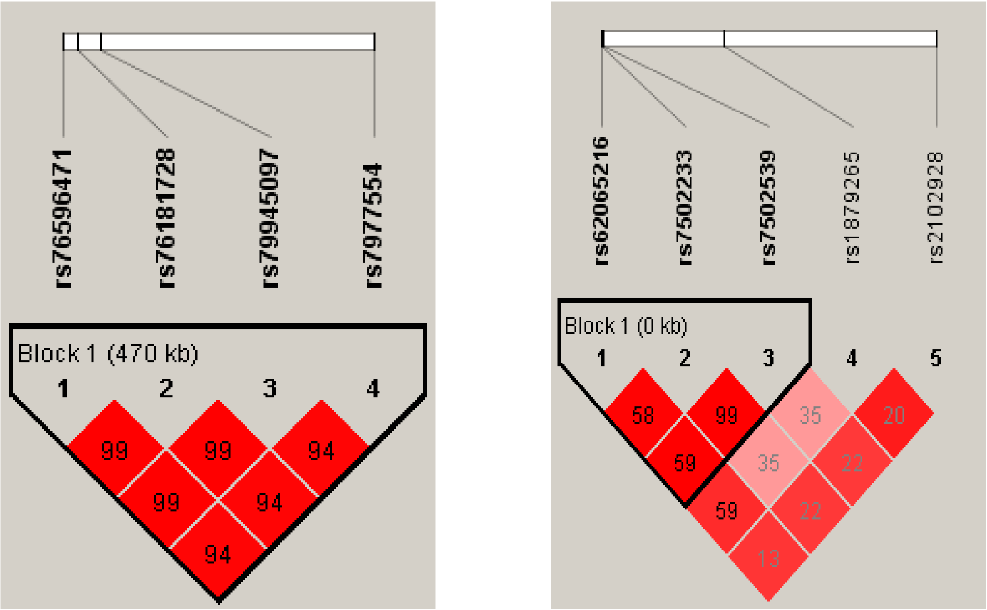

(a) Chromosome 12 (b) Chromosome 17

Figure S3. Linkage disequilibrium plot.

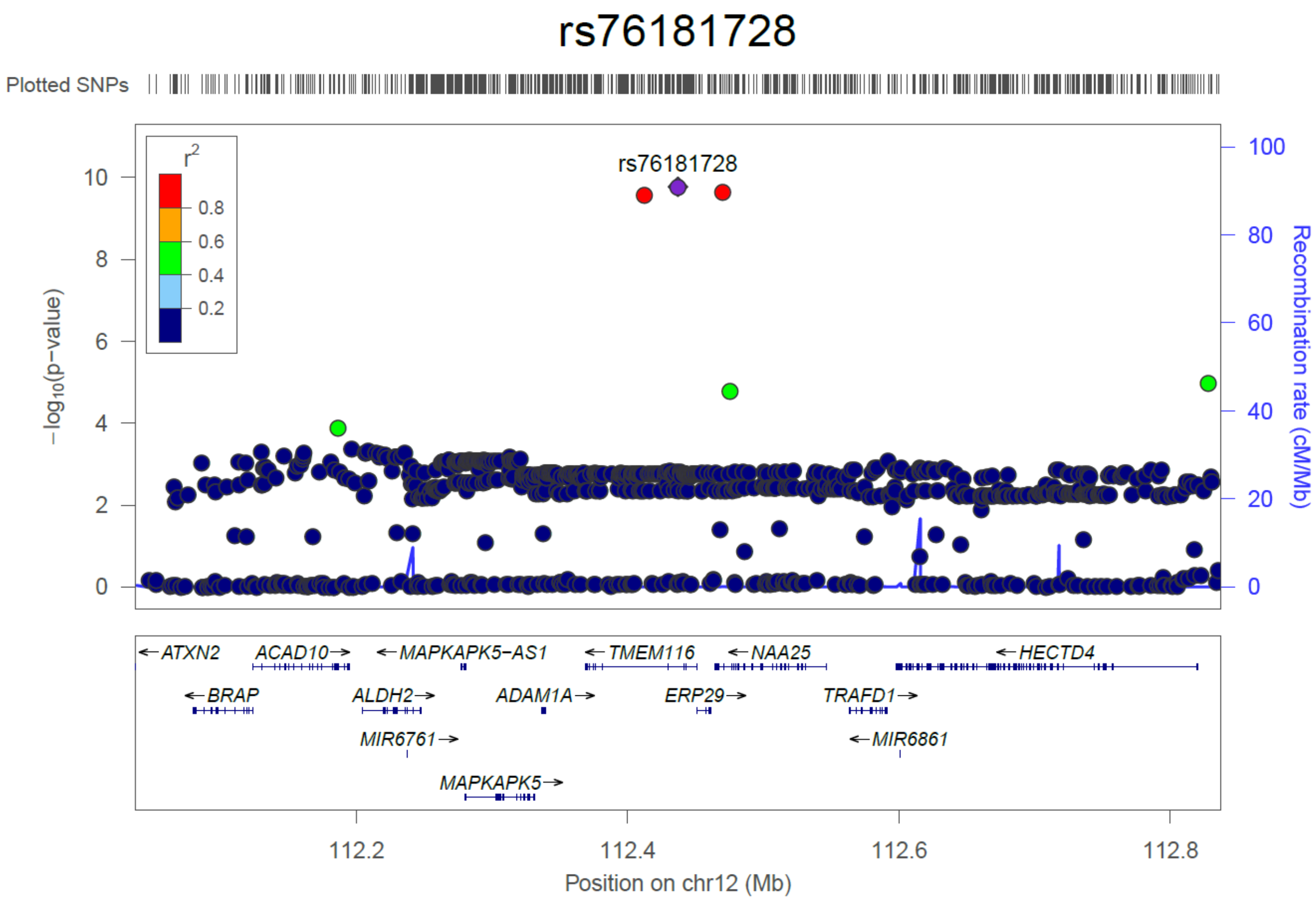

Figure S4. Regional plot for the top SNP rs76181728 associated with the log-transformed NLR.
